# Patient outcomes, patient experiences and process indicators associated with the routine use of patient-reported outcome measures (PROMs) in cancer care: a systematic review

**DOI:** 10.1007/s00520-020-05695-4

**Published:** 2020-09-02

**Authors:** Caitlin Graupner, Merel L. Kimman, Suzanne Mul, Annerika H. M. Slok, Danny Claessens, Jos Kleijnen, Carmen D. Dirksen, Stéphanie O. Breukink

**Affiliations:** 1 Department of Surgery, Maastricht University Medical Center, P. Debyelaan 25, 6229 HX Maastricht, The Netherlands; 2 School for Oncology and Developmental Biology (GROW), Maastricht University, Maastricht, The Netherlands; 3 Department of Clinical Epidemiology and Medical Technology Assessment, Maastricht University Medical Center and Care and Public Health Research Institute (CAPHRI), Maastricht, The Netherlands; 4 Department of Family Medicine, Care and Public Health Research Institute (CAPHRI), Maastricht University, Maastricht, The Netherlands; 5 Kleijnen Systematic Reviews Ltd, York, UK; 6 Care and Public Health Research Institute (CAPHRI), Maastricht University, Maastricht, The Netherlands

**Keywords:** Patient-reported outcome measures, PROM, Cancer, Patient-reported outcomes

## Abstract

**Purpose:**

In current cancer care, there is a growing debate about the value of using patient-reported outcome measures (PROMs) in daily clinical follow-up. A systematic review of the literature was conducted to assess the evidence of the effectiveness of the routine use of PROMs in daily cancer care in terms of patient outcomes, patient experiences and process indicators and to identify the effect of giving feedback about PROM findings to patients and/or health care professionals (HCPs).

**Methods:**

A systematic search was performed. Studies were eligible for inclusion when they (1) used a PROM as an intervention, with or without feedback to patients and/or HCPs, compared with not using a PROM, and (2) used a PROM as an intervention with feedback to patients and/or HCPs, compared with using a PROM without giving feedback to patients and/or HCPs.

**Results:**

After screening of 8341 references, 22 original studies met the inclusion criteria. Most studies found a positive effect on survival, symptoms, HRQoL and patient satisfaction. In general, using feedback to patient and/or HCPs about the PROM results led to better symptom control, HRQoL, patient satisfaction and patient-doctor communication. The majority of included studies had insufficient power to detect significant differences in the outcomes assessed.

**Conclusion:**

This review shows that predominantly positive findings were found in the use of a PROM in daily cancer care. Additionally, more positive effects were seen when feedback is provided to patient and/or health care professionals, and it is thus highly recommended that this is always done.

## Introduction

With improved cancer treatment modalities, the number of cancer survivors is rising [1]. For years, clinicians only focussed on traditional oncological outcomes, such as mortality and morbidity, in order to evaluate treatment effectiveness [2]. While survival and detection of recurrence are still the main pillars of cancer care follow-up, monitoring patient-centred outcomes, such as health-related quality of life (HRQoL), independence or fatigue, is now an accepted component of follow-up care [3]. This shift is also reflected in several oncological outcome sets of International Consortium of Health Outcomes Measurement (ICHOM) [4–6].

It is well known that cancer patients may have a high symptom burden which is caused either by the disease itself or their treatment modalities [7–9]. Underreporting of these symptoms by patients and underassessment by caregivers could result in under recognition and under treatment of these symptoms in daily oncological practice [10, 11]. By using patient-reported outcome measures (PROMs), the patient’s perception of the impact of their condition or treatment on their quality of life can be evaluated and, if necessary, acted upon [12]. PROMs can focus on perceived needs, symptoms, response to treatment, undesirable side effects, effect on function or other aspects of the treatment that matter to patients and their families. Besides, PROMs can be used in shared decision-making throughout the entire process from diagnosis to follow-up [10, 13, 14]. Aggregated PROM data may also serve other purposes, such as quality improvement processes, clinical research and internal and external benchmarking [15]*.*

In current cancer care, there is a growing debate about the additional value of using PROMs in daily clinical follow-up. Several oncological studies indicated that the systematic use of PROMs is associated with improved patient-physician communication [16], higher patient satisfaction [12] and improvement of patient symptom control [17]. A review by Kotronoulas et al. (2014) focused on PROM use in cancer care and investigated the effect of PROMs with and without individualized management plan [18]. The outcomes of this review focused on patient outcomes, process of care and health service outcomes. Their search was performed in 2013, and they included 26 studies in their review. They concluded that using PROMs increased the discussion of patient outcomes during consultations and that PROMs were associated with an improved symptom control and patient satisfaction. A recent systematic review by Ishaque et al. (2019) investigated the effectiveness of PROMs as an intervention to support the representation of patient values and preferences in clinical encounters in oncology (*n* = 12) and non-oncology settings (*n* = 10) [19]. They described overall positive findings in favour of the PROM intervention, especially when PROM results were shared with clinicians.

Opponents declare that the evidence of the additional value of PROMs is limited. Completing PROMs may be stressful and time-consuming for patients. A known barrier of using PROMs in daily care is the amount of extra administrative efforts experienced by health care professionals (HCPs) [20–22]. A properly functioning IT system could solve some of these extra efforts, yet this is often lacking [23].

The aim of this systematic review is to provide an up-to-date evidence synthesis of the effectiveness of routine use of PROMs in daily cancer care, in terms of patient outcomes and experiences. The secondary objective is to identify the effect of giving feedback about PROM findings to patients and/or health care professionals compared with PROM use without any feedback.

## Methods

### Search

A systematic search was performed in September 2018 in MEDLINE, EMBASE, Cochrane Library Database, PubMed and CINAHL. Studies published within the last 20 years (1998–2018) were included. An update of the search was performed in December 2019. The search terms were chosen in such a way that any description that could resemble or relate to the use of PROMs within oncology would be discovered by the search (Appendix Table 5). Additional articles were identified by examining the reference lists of reviewed articles. No language restrictions were applied. Studies included in systematic reviews and meta-analyses were checked for eligibility.

### Study selection criteria

Preferred Reporting Items for Systematic Reviews and Meta-Analyses (PRISMA) guidelines were followed throughout the review process [24]. Study selection consisted of a two-phase process performed by three researchers (CG, LM and SM). First, titles and abstracts were screened, and potentially eligible articles were retrieved independently by two researchers (CG and LM). Second, full-text reports were read by two authors (CG and SM), independently, to determine eligibility of the studies. Furthermore, the reference lists of eligible studies were checked for any missing studies. In case of disagreement in one of the two phases, a third reviewer (MK) was consulted until agreement was reached.

For the first aim, studies were eligible for inclusion when they used a PROM as intervention, with or without feedback to patients or health care professionals, compared with not using a PROM. For the second aim, studies were eligible when they used a PROM as intervention with feedback to patients or health care professionals, compared with a control group in which PROMs were used without giving feedback to patients or health care professionals about the results. All types of cancer patients were included, and no specific care settings were in- or excluded. All clinical trials and observational studies with a control group were included.

Studies were excluded if they were a validation study of a PROM, if the use of the PROM was to evaluate another intervention (e.g. treatment or follow-up strategy), when the study compared PROM intervention modalities (e.g. PROM A vs PROM B) or when the study focussed on children (< 18 years).

### Risk of bias and methodological quality evaluation

Risk of bias evaluation of all included studies was performed by two reviewers using the Cochrane Collaboration Risk of Bias Tool [25].

### Data synthesis and analysis

Data extraction, synthesis and analysis were performed by two independent reviewers. The following study characteristics were extracted from each study: author, year, setting, study population, number of participants, intervention, control, method of data collection, education in interpretation yes/no, feedback received by patient or health care professionals yes/no, patient outcomes and patient experiences. Feedback received by patients could be a summary of results or a treatment advice based on the results of the PROM.

Extracted outcomes and experiences were synthesized in a narrative matter and categorized into one of five categories: survival/mortality, symptoms/morbidity, health-related quality of life (HRQoL), patient satisfaction and process of care (number of discussed topics, duration consultation, emergency room visits, management/treatment actions, patient-doctor communication).

## Results

### Study characteristics

**Fig. 1:**
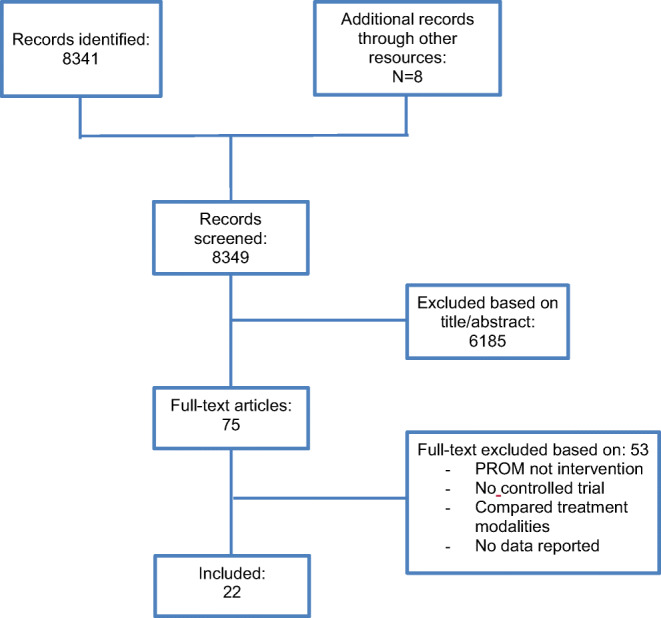
Flow diagram of study identification and selection

After removal of duplicates, 8341 references were identified through the initial search. An additional eight references were added by checking the reference list of previously published literature reviews [18, 19]. Of 75 references eligible for full-text screening, 22 met inclusion criteria and were included in the final analysis (Fig. 1).

Of the included studies, 19 (86%) were randomized controlled trials and three (14%) were sequential two-arm cohort studies. Most studies (*n* = 20, 90%) were conducted in an outpatient clinic setting [16, 17, 26–43]. One study [44] was performed at a hospice and one study at an inpatient clinic [45]. Patients with various cancer types (including lung, breast, colorectal, gynaecologic, prostate, head and neck, lymphatic and prostate) and treatment modalities participated in the individual studies. The number of included patients ranged between 43 and 766 across the studies (Tables 1 and Appendix Table 6).

**Table 1:** Characteristics of included studies

	Number of studies	Portion of total (%)
Setting		
- Outpatient clinic	20	90
- In- and outpatient clinic	1	5
- Hospice	1	5
Study design		
- Randomized controlled trial	19	86
- Cohort study	3	14
Types of cancer		
- Various cancer types (including breast, colorectal, lung, gynaecologic, head and neck, etc.)	16	72
- Lung	4	18
- Lymphatic	1	5
- Prostate	1	5
Number of patients in included studies		
- 1–100	5	22
- 101–250	10	45
- 251–500	4	18
- > 500	3	14
Control group received		
- No PROM (care as usual)	15	68
- PROM	7	32

^1^Percentages are rounded to the nearest percent

The majority of the studies were conducted in the USA (*n* = 8), followed by the UK (*n* = 5) and the Netherlands (*n* = 3). Great diversity was seen in types of PROMs (*n* = 20), and several studies used more than one PROM (Table 2). The European Organisation for Research and Treatment of Cancer Quality of Life C30 (EORTC QLQ-C30) (*n* = 6), general symptoms on a numeric scale (*n* = 6) and the Hospital Anxiety and Depression Scale (HADS) (*n* = 3) were the most commonly used PROMs in the included studies.

**Table 2:** Characteristics of intervention

	Number of studies	Portion of total (%)
PROMs		
- European Organisation for Research and Treatment of Cancer, Quality of Life - Cancer 30 (EORTC QLQ-C30 )	6	17
- European Organisation for Research and Treatment of Cancer, Quality of Life - Lung 30 (EORTC QLQ-LC13 )	3	9
- European Organisation for Research and Treatment of Cancer, Quality of Life - Breast 23 (EORTC QLQ-BR23 )	1	3
- European Organisation for Research and Treatment of Cancer, Quality of Life - Colorectal 38 (EORTC QLQ-CR38 )	1	3
- General symptoms (on a numeric scale)	7	20
- Hospital Anxiety and Depression Scale (HADS)		
- Symptom Tracking And Reporting (STAR)	3	9
- Supportive Care Needs Survey (SCNS)	1	3
- Functional Assessment of Cancer Therapy – General (FACT-G)	1	3
- Functional Assessment of Cancer Therapy – Prostate (FACT-P)	1	3
- MD Anderson Symptom Inventory (MDASI)	1	3
- Common Toxicity Criteria Adverse Events (CTCAE)	1	3
- Chemotherapy Symptom Assessment Scale (CSAS)	1	3
- Palliative Performance Scale (PPS)	1	3
- Memorial Symptom Assessment Scale (MSAS)	1	3
- Hospice Quality of Life (HQLI)	1	3
- Center for Epidemiological Studies-Depression (CES-D)	1	3
- Spiritual Needs Inventory (SNI)	1	3
- Short Portable Mental Status Questionnaire (SPMSQ)	1	3
- Therapy Related Symptom Checklist (TRSC)	1	3
Location of data collection		
- Home	7	32
- Outpatient clinic	12	54
- In- and outpatient clinic	1	5
- Not reported	2	9
Methods of data collection		
- Paper	6	27
- Electronic	11	50
- Paper and electronic	1	5
- Telephone	2	9
- Not reported	2	9
Feedback received by:		
- Health care professional	15	68
- Patient and health care professional	6	27
- Not reported	1	5
Education in interpretation for health care professional		
- Yes	6	27
- No	14	64
- Not reported	2	9

^1^Percentages are rounded to the nearest percent

### Risk of bias

**Fig. 2:**
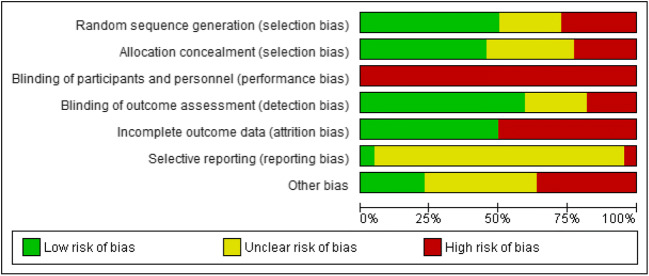
Risk of bias presented as percentages across all included studies using Cochrane Risk of Bias Tool

Figure 2 summarizes the risk of bias of the included studies. Risk of bias (selection, performance, detection, attrition, reporting and other types of bias) was assessed using the Cochrane Risk of Bias Tool [25]. For the risk of bias assessment of each individual study, see Appendix Table 7. Random sequence generation risk of bias was as expected high in the three non-randomized controlled trials (RCTs) [30, 38, 42]. Allocation concealment was maintained in nine studies; in seven studies, this was not reported; and in five studies, there was a high risk of bias. All included studies were rated as high risk regarding performance bias as blinding of participants and personnel was not possible due to the nature of a PROM intervention. Twelve studies (57%) were rated as low risk for detection bias. Ten studies (48%) reported high rates of drop-out or loss to follow-up and were therefore rated as high risk of bias due to incomplete outcome data. Reporting bias was unclear in almost all studies.

### PROM as intervention, with or without feedback to patients or health care professionals, compared with not using a PROM

Of the 22 included studies, 15 studies were identified that compared the use of a PROM as the intervention to no PROM intervention [16, 26, 29–33, 36, 38, 43, 44]. Of these fifteen studies, in one study, the intervention was merely the use of a PROM [31]. In 14 studies, the use of the PROM was supplemented with feedback about the results [16, 26, 29, 30, 32, 33, 36, 38–44]. In case feedback was provided, it was provided either to the HCP, the patient or both. In seven studies, the feedback was only available to the HCPs [36, 38–42, 44]. In five studies, the feedback was provided to both patients and HCPs [16, 26, 29, 30, 32]. In two studies, the results were available for HCPs if patients proactively shared the feedback with them (Table 3) [33, 43].

**Table 3:** Main findings and outcome assessment comparing PROM as intervention, with or without feedback to patients or health care professionals, to not using a PROM

Author and year	Survival/mortality	Morbidity	Health-Related Quality of Life (HRQoL)	Patient satisfaction	Process indicators
Basch et al. 2016 [26]	Overall survival after 1 year was higher in IG than CG; 75% vs 69%, *p* = 0.05. Difference was more pronounced among computer-inexperienced participants; 74% vs 60%, *p* = 0.02). Quality-adjusted survival (in months) observed in one year was higher in IG than CG; mean 8.7 months vs 8.0 months, *p* = 0.004	NR	More patients showed improved HRQoL in IG than CG (34% vs 18%), and fewer patients reported declined HRQoL scores (38% vs 54%)	NR	Patients in IG were less frequently admitted to the emergency room (34% vs 41%, *p* = 0.02) or hospitalized (45% vs 49%, *p* = 0.08) Patient in IG received longer active chemotherapy compared with CG; mean 8.2 months (0–49 months) vs mean 6.3 months (0–41 months), *p* = 0.002
Davis et al. 2013 [29]	NR	NR	HRQoL did not significantly differ between groups. Mean scores were IG 92.5 (SD 12.3), CG 94.8 (SD 11.3); *p* > 0.10.	85% of patients (*n* = 60) endorsed that all patients would benefit from an automated monitoring system to routinely assess their symptoms/HRQoL	Doctor/patient communication did not change over time in both groups
Detmar et al. 2002 [16]	NR	NR	HRQoL did not significantly differ between groups on any of the subscales. On two subscales, a greater percentage of patients in IG compared with CG showed improvement over time (defined as 0.5 SD unit or greater change). Mental health (43% vs 30%; *p* = 0.04) and role functioning (22% vs 11%; *p* = 0.05)	Patient satisfaction was high in both groups. The degree of received emotional support was higher in IG than CG; mean 4.3 (SD 0.72) and 4.0 (SD 0.89); *p* = 0.05 Almost all patients (97%) reported that the HRQoL profiles provided an accurate picture of their functioning and well-being. 79% believed it enhanced their physicians’ awareness of their health problems	Doctor/patient communication was higher in IG than CG. Mean scores were 4.5 (SD 2.3) and 3.7 (SD 1.9), respectively; *p* = 0.01 HRQoL topics were discussed more often in IG than CG No differences were seen in patient management actions. Mean number of actions undertaken were are 0.6 in IG and 0.5 in CG A higher percentage of patients in IG than CG received counselling from their physician on how to manage their health problems 23% vs 16% *p* = 0.05
Hilarius et al. 2008 [30]	NR	NR	HRQoL did not significantly differ between groups. Specific data not supplied by authors	Patient satisfaction was high in both groups. No statistically significant group differences were observed. Specific data not supplied by authors	The mean composite communication score regarding HRQoL was higher in IG than CG (*p* = 0.009). Mean scores were 4.8 (SD 3.3) and 3.8 (SD 2.3), respectively In IG, HRQoL topics were discussed more frequently than in CG No differences were seen in patient management activities (referral, medication prescription, test ordering, modification chemotherapy) between groups
Hoekstra et al. 2006 [31]	NR	After 2 months, f/u all symptoms, except coughing, were less prevalent in IG than in CG (range prevalence − 2.1 to − 24.3%). Only coughing was more prevalent in IG (14.9%).Constipation, vomiting and sleeplessness showed relatively large differences (24%, 18% and 18%, respectively) in favour of IG, but only constipation and vomiting were statistically significant (no specific data were given by authors) Fatigue, lack of appetite, shortness of breath and nausea were rated less severe in IG, but this was not statistically significant Severity of pain, coughing, sleeplessness and diarrhoea were rated equally severe in both groups. Only constipation and vomiting were significantly experienced as more severe (*P* < 0.05)	NR	NR	NR
Kearney et al. 2009 [32]	NR	More patients in CG reported fatigues than in IG. 81.3% vs 67.3% respectively; odds ratio = 2.29 (95% CI 1.04 to 5.05) *p* = 0.040 Hand-foot syndrome was less often present in the CG compared with IG. 12.2% vs 24.0%, respectively, odds ratio = 0.39 (95% CI 0.17 to 0.92) *p* = 0.031 No differences were seen in vomiting, nausea, diarrhoea and sore mouth/throat More severe hand-food syndrome and distress were seen in IG compared with CG: mean 0.46 (SD 0.64) vs 0.22 (SD 0.49); *p* = 0.033) and mean 0.30 (SD 0.45) vs 0.16 (SD 0.34); *p* = 0.028, respectively Other symptoms showed no significant differences in severity and distress between groups	NR	NR	NR
Matsuda et al. 2019 [43]	NR	NR	HRQoL did not significantly differ between groups. An effect size of 7.39 (95% CI − 6.39 to 21.17; *p* = 0.285) was seen in favour of the IG over time	NR	NR
McMillan et al. 2011 [44]	NR	In both groups, depression scores declined significantly over time (*p* = 0.023). Decline of depression scores was more present in IG than in CG (*p* = 0.027) No between-group differences were seen in distress scores and spiritual needs Specific data not supplied by authors.	In both groups, HRQoL improved significantly over time (*p* < 0.001), but no differences between IG and CG were seen Specific data not supplied by authors	NR	NR
Mills et al. 2009 [33]	NR	NR	IG had a lower overall and lung-specific HRQoL than the CG. Overall scores declined 6.6 (SD 12.5) in IG and inclined 0.2 (SD 15.7) in CG; *p* = 0.10 Lung-specific scores declined 6.3 (SD 14.9) in IG and inclined 3.5 (SD 18.4) in CG; *p* = 0.05.	Both groups reported high levels of satisfaction with their care. CG reported slightly higher satisfaction, and no significant associations were identified	Little participants of IG discussed their results with the HCP (23%, *n* = 13). Patient in IG discussed fewer topics with HCP’s than CG, no statistically significant differences were seen
Rosenbloom et al. 2007 [36]	NR	NR	No significant differences were observed in HRQoL across the three study groups (*p* > 0.05) Mean score of IG, ACG and CG were 115.8 (SD 22.9), 113.3 (SD 24.5) and 112.2 (SD 21.4), respectively	No significant differences were observed in general satisfaction and satisfaction with communication across the three study groups (*p* > 0.05) General satisfaction mean scores of IG, ACG and CG were 22.4 (SD 4.2), 23.1 (SD 4.2) and 24.4 (SD 4.1), respectively General satisfaction mean scores of IG, ACG and CG were 21.2 (SD 2.8), 21.1 (SD 3.0) and 20.8 (SD 3.2), respectively	Change in clinical treatment did not significantly differ between groups
Taenzer et al. 2000 [38]	NR	NR	Four HRQoL-subscales (emotional, cognitive, social and global functioning) did not significantly differ between groups. CG scored better on two HRQoL subscales: physical functioning (*p* < 0.05) and role functioning (*p* < − 0.01) compared with the IG There was a high degree of variation in scores, particularly on symptom scales, indicating a wide range of QoL	Satisfaction did not significantly differ between groups (*P* > 0.05). Overall levels of patient satisfaction were high	In IG, HRQoL topics were more frequently discussed than in CG. Number of topics mean 6.4 (SD 4.1) vs 2.5 (SD 2.9); *p* < 0.01 A higher percentage of taken actions on identified HRQoL topics was seen in IG than CG (73% vs 68.5%)
Takeuchi et al. 2011 [39]	NR	NR	NR	NR	In IG and ACG, more symptoms were discussed than in CG; *p* = 0.040 and *p* = 0.08, respectively Number of discussed symptoms was higher for all groups at the first consultation compared with the third consultation; *p* = 0.004
Velikova et al. 2004 [40]	NR	NR	HRQoL was higher in IG compared with CG and ACG; *p* = 0.006 and *p* = 0.80, respectively. HRQoL was higher in ACG compared with CG; *p* = 0.01 Specific data not supplied by authors	NR	In IG, more HRQoL topics were discussed compared with CG; mean number of topics discussed were 3.3 vs 2.7 Consultations did not prolong in IG; mean time per consultation 12.6 min in IG vs 12.8 min in CG
Velikova et al. 2010 [41]	NR	NR	NR	86% (*n* = 85) of patients in IG perceived that PROMs were useful to tell physicians how they were feeling compared with 29% (*n* = 34) in CG. Between 79 and 89% of all patients rated their quality of care as ‘very good’ or ‘excellent’	Communication in the IG was rated better than in the CG; *p* = 0.03. No significant differences were seen in communication between IG and ACG; *p* = 0.16
Williams et al. 2013 [42]	NR	NR	HRQoL increased by 3.31 points in the IG (*p* = 0.12), whereby an increase of 3.0 points was seen as clinically significant No specific data was supplied for CG by authors	NR	In IG, more symptoms were documented and managed than in CG; mean number of symptoms: 3.76 (*p* < 0.001) The number of symptoms documented and managed increased by 0.76 for each cancer stage greater than stage I (*p* < 0.03)

*ACG* attention-control, *CG* control group, *ES* effect size, *HCP* Health Care Professionals, *HRQoL* Health-Related Quality of Life, *IG* intervention group, *NR* not reported, *SD* Standard Deviation

#### Patient outcomes—survival/mortality

Only one study included survival as an outcome [26]. Basch et al. (2016) found that overall survival after 1 year was 76% in the PROM group versus 68% in the non-PROM group (*p* = 0.05). The study identified two important subgroups: computer-experienced and computer-inexperienced patients. Only one study included survival as an outcome [26]. Notably, in the subgroup of computer-inexperienced patients within the intervention group, the survival rate was significantly higher compared with the computer-inexperienced patients within the control group (*p* = 0.02). For the patients who were computer-experienced, no significant difference in survival was identified between the intervention and control group (*p* = 0.45). The authors suggested that computer-inexperienced patients may have less-developed health communication skills and thereby benefit more from a structured program that incorporates self-reporting via PROMs.

#### Patient outcomes—morbidity and symptoms

Three studies evaluated symptoms as an outcome [31, 32, 44]. In the study of McMillan et al. (2011), patients with various cancer diagnoses who were admitted in a hospice filled out five PROMs that focused on symptoms, spiritual needs and HRQoL (i.e. PPS, MSAS, HQLI-4, CES-D, SNI, SPMSQ). The only outcome with a significant difference between the intervention group and control group was depression, as measured by the CES-D. While the depression scores declined significantly (*p* = 0.023) over time in both the intervention and control group, indicating an improvement in mental health, decline was significantly larger in the intervention group than in the control group (*p* = 0.027) [44].

In the study by Hoekstra et al. (2006), cancer patients receiving palliative treatment were randomized to either completing a symptom-based PROM (ten symptoms on a numeric 1–10 scale) every week at home or not completing a PROM. The symptoms evaluated were fatigue, pain, lack of appetite, shortness of breath, coughing, sleeplessness, nausea, constipation, diarrhoea and vomiting. It is unclear whether results were reported to the HCP and the patients. Significant differences were only identified for vomiting and constipation. The prevalence of these symptoms was lower in the intervention group [31].

Kearney et al. (2009) compared the use of a PROM (CTCAE and Chemotherapy Symptom Assessment Scale integrated into one questionnaire) for 14 days after a cycle of chemotherapy in breast, lung and colorectal cancer patients to care as usual without a PROM and found that the intervention group had a higher prevalence of hand-foot syndrome. Furthermore, the severity of hand-foot syndrome and associated levels of distress were also significantly higher. Other symptoms reported in the PROM did not differ significantly between the intervention and control group [32].

#### Patient outcomes—HRQoL

Studies performed by Basch et al. (2016), Velikova et al. (2004) and Williams et al. (2013) showed a positive effect on HRQoL when using a PROM compared with no PROM [26, 40, 42]. In these studies, patients in the intervention group reported a significant and clinically relevant improvement in HRQoL over time. Six studies did not identify significant difference between HRQoL scores between the intervention and control group [16, 29, 30, 36, 43, 44]. Mills et al. (2009) and Taenzer et al. (2000) found a negative effect when using a PROM [33, 38]. In the study of Mills et al. (2009), patients in the control group (i.e. no PROM) scored better on a lung-specific HRQoL scale, which included physical well-being, social/family well-being, emotional well-being, functional well-being and seven lung cancer-specific symptoms (*p* = 0.04) [33]. In the study of Taenzer et al. (2000), patients in the control group scored better on two specific HRQoL sub-domains, physical functioning and role functioning (*p* < 0.05 and *p* < 0.01, respectively). In all other HRQoL domains, no significant differences were found between the PROM intervention and control group [38]. A lower reported HRQoL in the intervention group may be explained by the increased attention to HRQoL domains, resulting in more recognition and reporting of specific symptoms.

#### Patient experiences—patient satisfaction

In three studies, patients expressed the usefulness of a PROM [16, 29, 41]. They stated that PROMs were useful to tell their physician how they were feeling and that they represented an accurate representation of their functioning and well-being. The majority of patients, 79, 85 and 86%, respectively, were confident in that a PROM increased the awareness of their physician regarding their HRQoL and symptoms [16, 29, 41].

Four studies described no significant difference in patient satisfaction between patients completing a PROM and those who did not complete a PROM as part of their cancer care [30, 33, 36, 38].

#### Process indicators

The study performed by Basch et al. (2016) reported on emergency visits and hospital admissions. The intervention group (i.e. patients receiving a PROM with feedback) reported statistically significant fewer emergency visits and hospital admissions than the control group (i.e. no PROM)[26].

Evaluation of doctor-patient communication was described in four studies comparing a PROM intervention to no PROM [16, 29, 30, 41]. In three studies, doctor-patient communication was rated better using a PROM [16, 30, 41], and one study [29] did not find any differences in the doctor-patient communication between the intervention and control group. In the study performed by Hilarius et al. (2008), more HRQoL topics were discussed in the intervention group than in the control group, but this did not lead to differences in patient management activities (e.g. referral, medication prescription, test ordering and modification chemotherapy) between both groups [30].

### PROM as intervention with feedback to patients or health care professionals, compared with a control group in which PROMs were used without giving feedback to patients or health care professionals about the results

Seven studies were identified that compared use of a PROM with feedback to patients or health care professionals to use of a PROM without feedback to patients or health care professionals [17, 27, 28, 34, 35, 37, 45] (Table 4).

**Table 4:** Main findings and outcome assessment comparing PROM as intervention with feedback to patients or health care professionals, compared with a control group in which PROMs were used without giving feedback to patients or health care professionals about the results

Author and year	Survival/mortality	Morbidity	Health-Related Quality of Life (HRQoL)	Patient satisfaction	Process indicators
Berry et al. 2011 [27]	NR	NR	NR	NR	If symptoms or HRQoL issues reached the alert-threshold in the IG, there was a 29% increase in the odd that these symptoms or HRQoL were discussed in consultation; odds-ratio 1.287( 95% CI 1.047 to 1.583) Length of clinic visits did not differ in length between groups. IG mean 30.3 (SD 17.9) min vs CG 31.7 (SD 18.8) min
Boyes et al. 2006 [17]	NR	Mean anxiety scores decreased in IG (6.83 at baseline to 4.80 at final f/u) more compared with CG (6.13 at baseline to 5.17 at final f/u); *p* = 0.09 Mean depression scores did not significantly differ between groups (*p* = 0.20). Mean depression scores in IG decreased from 4.98 to 4.20 (baseline to final f/u) and increased in CG from 3.84 to 3.91 (baseline to final f/u). No difference between IG and CG in moderate or high psychological needs (*p* = 0.82)	NR	NR	34 of 36 patients rated the PROM as easy to complete. 30 of 36 patients thought that using a PROMS was a good way for doctors to get information about patients’ well-being 3 of 20 patient in IG reported that their physician discussed the feedback report with them (*n* = 3). Two of four HCPs reported they discussed the feedback report with their patients. Patients in IG were less likely to report a bothersome symptom at a third visit when they already reported it at the second visit, compared with CG; OR = 2.8, *p* = 0.04
Cleeland et al. 2011 [28]	NR	A significant reduction of symptom threshold events was seen in both groups. The reduction rate was 19% in IG and 8% in CG. Rate ratio difference was 0.88 (95% CI 0.78 to 0.98) indicating IG approximately had 12% less symptom threshold events	NR	Patient in the IG were more satisfied with the intervention than patients in CG; mean score: 9.4 vs 8.4 respectively, *p* < 0.03. Patients in the IG rated the system more likely as easy to use; mean score 9.7 in IG vs 8.8 in CG, *p* < 0.01	NR
Mooney et al. 2014 [34]	NR	Symptom severity and distress scores did not significantly differ between groups (mean difference = 0.06; *p* = 0.58).	NR	79.0% of the patients in IG were quite or very confident that the automated system notified their physician of their symptoms 25.0% of the patients in IG agreed that the system helped their physician to decrease their symptoms	Unscheduled contacts did not significantly differ between groups (*p* = 0.73) Frequency of patient-initiated and physician-initiated contacts was similar (*p* = 0.14) Patients in CG talked somewhat more often about their symptoms (*n* = 79, 73.0%) at patient-initiated contacts than patients in IG (*n* = 64, 62.0%) There were more provider-initiated contacts that resulted in an office visit in the IG (*n* = 18, 17.5%) than in the CG (*n* = 10, 9.3%). In the provider-initiated contacts in IG symptoms were discussed more often (*n* = 14, 70.0%) than in the CG (*n* = 4, 33.0%), *p* = 0.10
Nicklasson et al. 2014 [35]	NR	NR	NR	NR	Emotional functioning was more discussed by doctors and patients in the IG than in the CG; mean 3.9 statements vs 2.4 statements; *p* = 0.015. Discussion of physical/role, social or cognitive functioning did not significantly differ between groups The sum of function-related statements by doctors and patients was higher in the IG compared with CG; mean 9.2 statements vs 6.9 statements; *p* = 0.0096 All symptoms (pain, dyspnea, fatigue, anorexia and other symptoms) were somewhat more discussed by doctors and patients in IG compared with CG (25.2 statements vs 24.5 statements), yet not significant, *p* = 0.36 Length of consultation was similar between groups. IG median 20 min vs CG median 22 min; range 8–60 min, *p* = 0.77) The number of diagnostic and therapeutic interventions per patient was statistically significant higher for emotional functioning (0.43 interventions vs 0.15 interventions; *p* = 0.0036), social functioning (1.17 interventions vs 0.74 interventions; *p* = 0.013) and dyspnea (1.08 interventions vs 0.53 interventions; *p* = 0.017), in the IG compared with the CG
Ruland et al. 2010 [45]	NR	Symptom distress declined over time in 10 of 19 (58%) symptoms in the IG (pain, eating/drinking, bowel/bladder, energy, sleep/rest, concentration/memory, activities of daily living/self-care and worries/concerns). Symptom distress declined in 2 of 19 symptoms in the CG (pain and worries/concerns). Discomfort, eating/drinking, sleep/rest and sexuality statistically differed between groups in favour of the IG Specific data not supplied by authors	NR	NR	17 of 19 symptoms showed a downward trend in patient needs for symptom management in the IG (*p* < 0.05) 14 symptoms in the CG showed an upward trend (6 of 19 were statistically significant *p* < 0.05) indicating that patients had greater needs for support to manage their problems over time Specific data not supplied by authors.
Strasser et al. 2016 [37]	NR	Symptom distress score between first and last visit was statistically lower in IG compared with CG. Mean difference between IG and CG: 5.70 (95% CI 1.96 to 9.43); *p* = 0.003	HRQoL was higher in IG than in CG. Mean difference between IG and CG 6.84 (95% CI − 1.65 to 15.33); *p* = 0.1	NR	A trend favouring IG (*p* = 0.06) was seen in symptom management performance. 71 (52%) patients in IG vs 40 (38%) patients in CG had symptom management interventions in visits where their symptom load was above a pre-set threshold. Specific data not supplied by authors

#### Patient outcomes—morbidity and symptoms

Five studies evaluated symptoms as an outcome [17, 28, 34, 37, 45]. In the study performed by Cleeland et al. (2011), an email was forwarded to the health care professionals in case a pre-set threshold of an alarming symptom (e.g. pain, distress, disturbed sleep, shortness of breath and constipation) was exceeded. Approximately 12% fewer emails regarding alarming symptoms were forwarded in the intervention group compared with the control group [28]. Ruland et al. (2010) found that in 75 leukaemia and lymphoma cancer patients who received feedback after completing a PROM, more symptoms had decreased compared with patients who had not received feedback about the findings of the PROM (ten of 19 symptoms vs two of 19 symptoms decreased). Of these ten symptoms, discomfort, eating/drinking, sleep/rest and sexuality were statistically significant in favour of the intervention group [45]. A favourable effect of using a PROM with feedback (compared with no feedback) was also seen in the study conducted by Strasser et al. (2016). The symptom distress score (including nine different symptoms rated on a 1–10 Likert scale) was significantly lower for the intervention than the control group over time (*p* = 0.003) [37]. In the study by Mooney et al. (2014), patients with various cancer types treated with chemotherapy were randomized to either reporting presence and severity of chemotherapy-related symptoms (rated on a 1–10 Likert scale) using an automated phone system with feedback to their physician or solely reporting chemotherapy-related symptoms using the identical automated phone system but without any feedback. No significant differences in symptom severity and distress scores were seen between the intervention and control group [34].

#### Patient outcomes—HRQoL

Only one article assessed HRQoL when comparing a PROM with or without feedback. Strasser et al. (2016) found a small, albeit significant, higher HRQoL in the group receiving a PROM with feedback. However, the difference between intervention group and control group was not considered clinically meaningful [37].

#### Patient experiences—patient satisfaction

Two studies focused on the comparison of PROMs with feedback and without feedback reported on patient satisfaction [28, 34]. In the study of Cleeland et al. (2011), higher levels of patient satisfaction were seen in the intervention group, compared with patients in the control group (*p* < 0.03). Mooney et al. (2014) found that 79% of patients were confident that the information they reported in the PROM would be noticed by their physician.

#### Process indicators

Mooney et al. (2014) reported that when health care professionals initiated the contact, more topics were discussed compared with when patients initiated contact [34]. In the study by Ruland et al. (2010), patients in the intervention group (i.e. PROM with feedback) and the control group (i.e. PROM without feedback) were asked to rate nineteen symptoms on a 1–10 Likert scale. In the intervention group, patients needed less symptom management in seventeen of nineteen symptoms. In six of these seventeen symptoms, significantly less symptom management or treatment was seen. In contrast to the intervention group, patients in the control group needed more symptom management in fourteen of nineteen symptoms, indicating that patients had greater needs for support in managing their symptoms. Significantly more symptom management was seen in six of these fourteen symptoms and all involved psychological needs—energy, sleep/rest, sexuality, mood/feelings, maintaining control over my situation and relationships [45]. In the study by Mooney et al. (2014), health care professionals treated both patients in the intervention group (i.e. receiving results of a PROM) and in the control group (i.e. not receiving results of a PROM). They found that the majority of health care professionals were satisfied with the PROM system and receiving alert reports, while 15% were not satisfied and did not read any of the received alert reports [34]. Berry et al. (2011) and Nicklasson et al. (2014) found no differences in consultation length between the group that received feedback on PROM results and the group that did not. Consultation time was not prolonged when health care professionals received feedback about PROM results.

## Discussion

Primarily, we found positive or insignificant results after the use of a PROM in daily cancer care. Only few studies found negative effects of using a PROM. There appears to be an association between using a PROM in daily cancer care and better outcomes in specific symptoms, HRQoL, patient satisfaction and patient-physician communication.

The first aim of this systematic review was to assess the effectiveness of PROMs and their effects on patient outcomes, patient experiences and process indicators. We identified 15 studies that compared the use of PROMs to not using PROMs. In twelve of the fifteen included studies, PROMs have shown a positive or no effect on survival, symptoms/morbidity, experienced HRQoL and patient satisfaction. Two studies reported a diminished experienced HRQoL.

HRQoL was the most commonly assessed outcome in studies evaluating PROM interventions. Eleven studies comparing a PROM versus no PROM used HRQoL as a primary or secondary outcome. While most studies (9/11) found that a PROM intervention led to better HRQoL scores or unchanged HRQoL scores, there were two studies that found that a PROM intervention resulted in reduced HRQoL scores. Raising awareness regarding specific HRQoL domains (e.g. physical well-being, social/family well-being, emotional well-being, functional well-being and role functioning) without sufficient feedback by health care professionals could lead to increased worrying and uncontrolled thought processes which in turn may result in a poorer HRQoL [33, 38, 46].

This review identified five studies that focused on symptoms as the outcome of the PROM intervention. Several symptoms (e.g. fatigue, constipation, vomiting) appeared to improve when completing symptom-based PROMs in the treatment trajectory, while others remained unaltered. A positive effect is likely to be related to contextual factors such as whether feedback to HCPs was provided and whether treatment strategies were then adapted to these findings. Most prominent, and somewhat inconsistent, were the findings regarding hand-foot symptoms, which were significantly more severe in the intervention group than in the control group in the study by Kearney et al. (2009) [32]. It is known that hand-foot symptoms are poorly assessed in routine cancer care [47]. It may be the case that participants in the study by Kearney et al. (2009) randomized to the intervention group were directed more to their hand-foot syndrome symptoms as part of the PROM intervention compared with the control group. It is expected that in the longer term, these symptoms would improve due to the attention paid to these symptoms.

In three studies, patient satisfaction was higher when using a PROM compared with no PROM, whereas all other studies showed no differences in patient satisfaction. Patient satisfaction scores are known to be prone to possible ceiling effects and may have limited responsiveness due to high levels of satisfaction before the intervention, leaving little room for improvement [48]. It may therefore be more desirable to approach this specific outcome in a qualitative manner. An evaluation of the experiences and satisfaction using, for example, individual interviews can give more insight into the actual improvements in this outcome due to the PROM intervention.

Some studies found that doctor-patient communication was rated higher by patients when a PROM was used [16, 30, 41]. This seems plausible since the PROM intervention resulted in more HRQoL topics being addressed during the consultation than in usual care. Basch et al. (2016) reported fewer visits to the emergency room or admissions in the hospital. A formal cost-utility was not performed by the authors, yet for future use and to promote uptake and implementation, it would be interesting to assess whether PROM interventions are cost-effective [26].

In the second aim of the review, we identified the effect of providing feedback to patients and/or health care professionals on the outcomes reported in PROMs and narrowed down the first aim by focusing only on providing feedback. Seven studies described the effect of giving feedback. Similar to the findings of Kotronoulas et al. (2014) and Ishaque et al. (2019), this review found that receiving feedback on the completed PROMs resulted in better symptom control (i.e. less symptom threshold events, diminished symptom distress scores and decreased depression and anxiety scores), less need for symptom management, higher patient satisfaction and improved patient-physician communication compared with control groups not receiving any feedback [17–19, 27, 28, 35, 45]. It is likely that patient-doctor communication improved because the PROM intervention identifies more HRQoL topics relevant to the patient that are subsequently discussed than in the usual care setting, for example, problems with sleeping or cognitive functioning. Creating awareness regarding experienced symptoms and HRQoL among both patients and health care professionals seems to be essential in retaining better patient outcomes and experiences. Providing feedback to patients by health care professionals can be helpful in increasing this awareness.

A known barrier of PROM implementation is time constraint experienced by health care professionals [37, 49]. However, this review did not identify any differences in consultation length between using feedback of a PROM and not using feedback [27, 35].

Precautions must be taken in interpreting the results of the individual studies and the evidence synthesis since many studies were at high risk of bias and had insufficient power to detect significant differences in the outcomes assessed. The majority of the studies focussed only on statistical significant differences (*p* values) and did not mention whether this difference was also clinical relevant and meaningful. Only Basch et al. (2016), Detmar et al. (2002), Mills et al. (2009), Velikova et al. (2004) and Williams et al. (2013) reported on both statistical and clinical significance.

Compared with the previous review by Kotronolous et al. (2014), who showed that using PROMs increased the discussion of patient outcomes during consultations and that PROMs were associated with an improved symptom control and patient satisfaction, five new studies were included. This was less than we had anticipated from the noticeable increase in PROM popularity. A possible explanation could be that PROMs are often used as an instrument to assess outcomes of treatment modalities, but not as an intervention in itself. Moreover, the focus of this review was narrower than the review by Kotronoulas et al. in the sense that we did not include personalized management plans or clinical interventions linked to the PROM intervention that could have influenced our outcomes of interest (e.g. survival, symptoms/morbidity, HRQoL, patient satisfaction and process indicators).

We have synthesized the data retrieved from the reviewed studies in a narrative manner, due to the fact that a large variety was seen in types of cancer patients, treatment, types of PROMs, application of PROMs, evaluated outcomes and whether feedback was given to patients and caregivers. All studies differed in follow-up time, applied PROM(s), intervention assessment and study population (Table 4). It was therefore impossible to perform any type of quantitative synthesis or compare individual studies to each other. Categorization of assessed outcomes was chosen in order to be able to analyse the results. With this heterogeneity in mind, no recommendations can be made to which PROM intervention is most effective, what follow-up duration would be optimal and what method of data collection should be used. The majority of the studies included a variety of people with cancer as the study population and did not analysed their data stratified by type of cancer or treatment. Hence, a ‘best practice’ for a cancer type or treatment modality cannot be extracted from this review. Nevertheless, the review has identified important insight into the current available evidence regarding PROMs and their role in daily cancer care.

Further research should focus on the evaluation of the interventions (i.e. PROMs) that had an effect on the various outcomes, with a focus on the content of the intervention, and the impact of the contextual environment in which the PROM is implemented, health care professionals’ attitudes and readiness to change and various implementation strategies on actual clinical outcomes.

## Conclusion

In general, predominantly positive or insignificant findings were found in the use of a PROM in daily cancer care. There appears to be a trend towards better outcomes in specific symptoms, HRQoL, patient satisfaction and patient-physician communication. More positive effect were seen when feedback is provided to patient and/or health care professional, and it is thus highly recommended that this is always done. This review provides evidence that the use of PROMs, especially when combined with feedback to patient and/or health care professional, can improve outcomes and experiences on an individual patient level.

## Figures and Tables

**Table 7:**
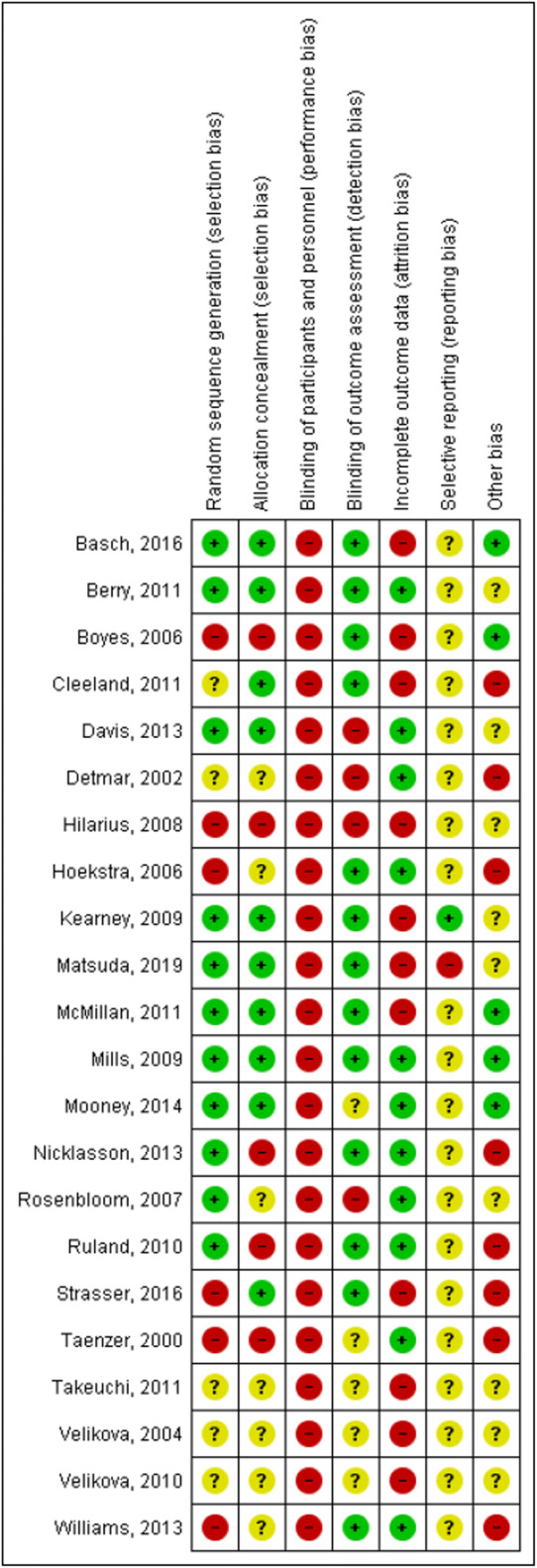
Risk of bias assessment of individual studies

## Appendix A

**Table 5:** Search strategy as conducted in Ovid Medline and EMBASE

Search terms used	
1. neoplasms/	
2. (neoplasm* or cancer* or carcinoma* or oncology* or malignan* or tumo?r* or leuk?emia* or sarcoma* or lymphoma* or blastoma* or melanoma*).ti,ab,kw.	
3. patient-reported outcome measure/	
4. (outcome* adj2 (measure* or tool* or assess* or score* or scale* or experience* or instrument* or questionnaire* or survey* or inventor*)).ti,ab,kw.	
5. (Patient adj3 (outcome* or measure* or tool* or assess* or score* or scale* or satisfaction or experience* or instrument* or questionnaire* or survey* or inventor*)).ti,ab,kw.	
6. ((self report* or self assess* or self monitor*) adj2 (outcome* or measure* or tool* or assess* or score* or scale* or satisfaction or experience* or instrument* or questionnaire* or survey* or inventor*)).ti,ab,kw.	
7. PROM.ti,ab,kw.	
8. PROMs.ti,ab,kw.	
9. (PREM or PREMs).ti,ab,kw.	
10. ((daily or routine* or consistent* or frequen* or regular* or standard or systematic) adj4 (use* or application or administ* or practice* or measure* or collection or assess* or utili?ation or monitor*)).ti,ab,kw.	
11. 1 or 2	
12. 3 or 4 or 5 or 6 or 7 or 8 or 9	
13. 11 and 12	
14. 10 and 11 and 12	

*ab* abstract, *adj* adjacency, *kw* keyword, *ti* title

## Appendix B

**Table 6:** Summary methodological characteristics included studies

Author and year of study	Setting/location	Patient population	No. of patients	Study design	Intervention (I)	Control (C)	Outcomes assessed	Method of collection	Feedback received by patient	Feedback received by HCP	Education in interpretation
Basch et al. 2016 [26]	Outpatient clinic, USA	Various metastatic cancer diagnosis	286 (I_1_); 253(C_1_); 155(I_2_);72(C_2_)	Stratified two-arm RCT	I_1_ Computer-experienced: Web-based PROM (STAR) before each outpatient visit at home, weekly between-visit completion was not mandatory but encouraged I_2_ Computer-inexperienced: web-based PROM (STAR) at outpatient visit Automatic email was send to HCP in case of deviating scores	Case as usual	Survival/mortality HRQoL	Electronic device at outpatient clinic and at home	Yes	Yes	No
Berry et al. 2011 [27]	Outpatient clinic, USA	Various cancer diagnosis treated with various treatments	327 (I); 333(C)	Two-arm RCT	Completion of PROM (SQLI) before f/u visit, feedback available for HCP	Completion of PROM (SQLI) no feedback available for HCP	Process indicators	Electronic tool at outpatient clinic	No	Yes	No
Boyes et al. 2006 [17]	Outpatient clinic, Australia	Various cancer diagnosis treated with various treatments	42(I); 38(C)	(pilot) two-arm non-RCT	Completion of PROM (physical symptoms, HADS, SCNS) before f/u visit. Feedback available for HCP	Completion of PROM (physical symptoms, HADS, SCNS) before f/u visit. No feedback available for HCP	Morbidity Process indicators	Electronic tool at outpatient clinic	No	Yes	Yes
Cleeland et al. 2011 [28]	Outpatient clinic, USA	Lung cancer of metastasis treated with thoracotomy	50(I); 50(C)	Two-arm RCT	Completion of PROM (MDASI) post-surgery for four week. Alarms were generated to treatment team when pre-set thresholds were exceeded	Completion of PROM (MDASI) post-surgery for four week. No alarms were generated.	Morbidity Patient satisfaction	Telephone based at home	No	Yes	No
Davis et al. 2013 [29]	Outpatient clinic, USA	Post-treatment prostate cancer survivors	49(I); 45(C)	Two-arm RCT	Completion of PROM (subscale FACT-P: PCS) before f/u visit	Care as usual	HRQoL Patient satisfaction Process indicators	Telephone based at home	Yes	Yes	Unclear
Detmar et al. 2002 [16]	Outpatient clinic, The Netherlands	Various cancer diagnosis, treated with chemotherapy	114(I); 100(C)	Two-arm cross-over RCT	Completion of PROM (EORTC QLQ-C30) in waiting room before f/u visit	Care as usual	HRQoL Patient satisfaction Process indicators	Paper tool at outpatient clinic	Yes	Yes	Yes
Hilarius et al. 2008 [30]	Outpatient clinic, The Netherlands	Various cancer diagnosis treated with chemotherapy	148(I); 150(C)	Sequential two-arm cohort	Completion of PROM (EORTC QLQ-C30, if applicable a condition specific EORTC module: QLQ-BR23, QLQ CRC38, QLQ-LC13) at outpatient visit	Care as usual	HRQoL Patient satisfaction Process indicators	Electronic device at outpatient clinic	Yes	Yes	Yes
Hoekstra et al. 2006 [31]	GP practice, The Netherlands	Various cancer diagnosis treated with palliative care	76(I); 83(C)	Two-arm RCT	Weekly PROM (The Symptom Monitor – 10 physical symptoms rated on a 0–10 numeric rating scale) completion in diary form	Care as usual	HRQoL	Paper tool at home	Unclear	Unclear	Unclear
Kearney et al. 2009 [32]	Outpatient clinic, UK	Breast, lung or colorectal cancer treated with chemotherapy	56(I); 56(C)	Two-arm RCT	Completion of PROM (CTCAE and chemotherapy Symptom Assessment Scale) days 1–14 post chemotherapy treatment. Information linked to an alert-system of clinician	Care as usual	HRQoL	Electronic tool at home	Yes	Yes	No
Matsuda et al. 2019 [43]	Outpatient clinic, Japan	Various cancer diagnosis treated with palliative care	21(I); 22(C)	Two-arm RCT	Daily completion of PROM (Care Notebook: 14-items cancer specific questionnaire)	Care as usual	HRQoL	Paper tool at home	Yes	If shared by patient.	Unclear
McMillan et al. 2011 [44]	Hospice, USA	Various cancer diagnosis	371 (I); 338(C)	Two-arm RCT	Weekly completion of PROM (PPS, MSAS, HQLI-4, CES-D, SNI, SPMSQ)	Care as usual	Morbidity HRQoL	Unclear	No	Yes	No
Mills et al. 2009 [33]	Outpatient clinic, UK	Inoperable lung cancer, all subtypes	57(I); 58(C)	Two-arm RCT	Weekly PROM (EORTC QLQ-C30, EORTC QLQ-LC13) in diary format	Care as usual	HRQoL Patient satisfaction Process indicators	Paper tool at home	Yes	If shared by patient.	No
Mooney et al. 2014 [34]	Outpatient clinic, USA	Various cancer diagnosis treated with chemotherapy	129(I); 121(C)	Two-arm RCT	Daily reporting of presence and severity of chemotherapy-related symptoms over the phone when receiving chemotherapy. Email alert to HCP when pre-set thresholds are exceeded	Daily report presence and severity of chemotherapy-related symptoms over the phone	Morbidity Patient satisfaction Process indicators	Telephone based at home	No	Yes	No
Nicklasson et al. 2013 [35]	Outpatient clinic, Sweden	Incurable lung cancer or mesothelioma	85(I); 88(C)	Two-arm RCT	Completion of PROM (EORTC QLQ-C30 and EORTC QLQ-LC13) before f/u visit. Feedback available for HCP	Completion of PROM (EORTC QLQ-C30 and EORTC QLQ-LC13) before f/u visit. No feedback available for HCP.	Process indicators	Electronic (I) and paper (C) tool at outpatient clinic	No	Yes	No
Rosenbloom et al. 2007 [36]	Outpatient clinic, USA	Advanced breast, lung or colorectal cancer treated with chemotherapy	69 (I); 73(AC); 71(C)	Three-arm RCT	Intervention: Completion of PROM before f/u visit (FACT-G and relevant disease specific subscale) followed by structured interview with research staff regarding patient’s response. Feedback available for HCP Attention-control: Completion of PROM (FACT-G and relevant disease specific subscale). Feedback available for HCP before f/u visit. No interview.	Care as usual	HRQoL Patient satisfaction Process indicators	Paper tool at outpatient clinic	No	Yes	No
Ruland et al. 2010 [45]	In- and outpatient clinic, Norway	Acute myelogenous leukaemia, lymphatic leukaemia, multiple myeloma, Hodgkin and non-Hodgkin disease	75(I); 70(C)	Two-arm RCT	Completion of PROM (19 symptoms rated on 0 to 4-scale) before every f/u visit and weekly for inpatient participants. Feedback available for HCP	No feedback available for HCP	Morbidity Process indicators	Electronic tool at in- and outpatient clinic	No	Yes	No
Strasser et al. 2016 [37]	Outpatient clinic, Switzerland	Various cancer diagnosis	145(I); 119(C)	Clustered two-arm RCT	Completion of PROM (nine symptoms on a visual analogue scale, nutrition intake, medication, body weight change, Karnofsky performance score) before f/u visit. Feedback available for HCP	Completion of PROM (nine symptoms on a visual analogue scale, nutrition intake, medication, body weight change, Karnofsky performance score) before f/u visit. No Feedback available for HCP	Morbidity HRQoL Process indicators	Electronic tool at outpatient clinic	No	Yes	No
Taenzer et al. 2000 [38]	Outpatient clinic, Canada	Lung cancer	27(I); 26(C)	Sequential two-arm cohort	Completion of PROM (EORTC QLQ-C30) before f/u visit	Care as usual	HRQoL Patient satisfaction Process indicators	Electronic tool at outpatient clinic	No	Yes	No
Takeuchi et al. 2011 [39]	Outpatient clinic, UK	Various cancer diagnosis treated with chemotherapy or biological therapy	100(I); 46 (AC); 52 (C)	Three-arm RCT	Intervention: completion of PROM (EORTC QLQ-C30, HADS) at outpatient clinic, feedback available for HCP Attention-control: completion of PROM (EORTC QLQ-C30, HADS) at outpatient clinic, no feedback available for HCP.	Care as usual	Process indicators	Electronic tool at outpatient clinic	No	Yes	Yes
Velikova et al. 2004 [40]	Outpatient clinic, UK	Various cancer diagnosis treated with various treatments	144(I); 70(AC); 72 (C)	Three-arm RCT	Intervention: completion of PROM (EORTC QLQ-C30, HADS) at outpatient visit, feedback available for HCP Attention-control: completion of PROM (EORTC QLQ-C30, HADS) at outpatient visit, no feedback available for HCP	Care as usual	HRQoL Process indicators	Electronic device at outpatient clinic	No	Yes, in intervention group	Yes
Velikova et al. 2010 [41]	Outpatient clinic, UK	Various cancer diagnosis treated with various treatments	144(I); 70(AC); 72 (C)	Three-arm RCT	Intervention: completion of PROM (EORTC QLQ-C30, HADS) at outpatient visit, feedback available for HCP Attention-control: completion of PROM (EORTC QLQ-C30, HADS) at outpatient visit, no feedback available for HCP	Care as usual	Patient satisfaction Process indicators	Electronic device at outpatient clinic	No	Yes, in intervention group	Yes
Williams et al., 2013 [42]	Outpatient clinic, USA	Various cancer diagnosis treated with chemo- or radiotherapy	58(I); 55(C)	Sequential two-arm cohort	Completion of PROM (TRSC) before f/u visit	Care as usual	HRQoL Process indicators	Paper tool at outpatient clinic	No	Yes	No

*AC* Attention-Control, *C* control, *CES-D* Centre for Epidemiological Studies-Depression, *CTCAE* Common Toxicity Criteria Adverse Events and Chemotherapy, *EORTC QLQ C30* European Organisation for Research and Treatment of Cancer, Quality of Life, Cancer 30, *EORTC QLQ-LC13* European Organisation for Research and Treatment of Cancer, Quality of Life, Lung Cancer 13, *FACT-G* Functional Assessment of Cancer Therapy–General, *f/u* follow-up care as usual excludes the use of a PROM, *HADS* Hospital Anxiety and Depression Scale, *HCP* Health Care Professionals, *HRQoL* Health-Related Quality of Life, *HQLI-14* Hospice Quality of Life Index, *I* Intervention, *MDASI* MD Anderson Symptom Inventory, *MSAS* Memorial Symptom Assessment Scale, *PPS* Palliative Performance Scale, *PROM* Patient-Reported Outcome Measure, *RCT* Randomized Controlled Trial, *RT* radiotherapy, *SCNS* Supportive Care Needs Survey, *SF-12* Short Form Health Survey, *SIPP* Screening Inventory of Psychosocial Problems, *SNI* Spiritual Needs Inventory, *SPMSQ* Short Portable Mental Status Questionnaire, *SQLI* Symptoms and Quality of Life Issues, *STAR* Symptom Tracking and Reporting, *TRST* Therapy Related Symptom Checklist, *UK* United Kingdom, *USA* United States

Table 6 contains a table in landscape format, and therefore it is added in an additional document *(Manuscript_PROMs in cancer care_Appendix B)*

## Abbreviations

PRO: Patient-reported outcome
PROM: Patient-reported outcome measure
HRQoL: Health-related quality of life
HCP: Health care professional
ICHOM: International Consortium of Health Outcomes Measurement
